# A Digital Companion, the Emma App, for Ecological Momentary Assessment and Prevention of Suicide: Quantitative Case Series Study

**DOI:** 10.2196/15741

**Published:** 2020-10-09

**Authors:** Margot Morgiève, Catherine Genty, Jérôme Azé, Jonathan Dubois, Marion Leboyer, Guillaume Vaiva, Sofian Berrouiguet, Philippe Courtet

**Affiliations:** 1 Department of Emergency Psychiatry and Acute Care, Lapeyronie Hospital, CHU Montpellier INSERM, Univ Montpellier, Neuropsychiatry: Epidemiological and Clinical Research Montpellier France; 2 ICM - Brain and Spine Institute, hôpital de la Pitié-Salpêtrière Paris France; 3 GEPS - Groupement d’Étude et de Prévention du Suicide Paris France; 4 LIRMM, UMR 5506, Montpellier University/CNRS Montpellier France; 5 Fondation Fondamental, hôpital Albert-Chenevier Créteil France; 6 CHU Lille, Hôpital Fontan, Department of Psychiatry Lille France; 7 Centre National de Ressources & Résilience pour les psychotraumatismes Lille France; 8 Université de Lille, CNRS UMR-9193, SCALab - Sciences Cognitives et Sciences Affectives Lille France; 9 EA 7479 SPURBO, Université de Bretagne Occidentale Brest France; 10 IMT Atlantique, Lab-STICC Brest France

**Keywords:** suicide, ecological momentary assessment, prediction, prevention, mobile health, mHealth, case reports, ecological momentary intervention

## Abstract

**Background:**

Many suicide risk factors have been identified, but traditional clinical methods do not allow for the accurate prediction of suicide behaviors. To face this challenge, *emma*, an app for ecological momentary assessment (EMA), ecological momentary intervention (EMI), and prediction of suicide risk in high-risk patients, was developed.

**Objective:**

The aim of this case report study was to describe how subjects at high risk of suicide use the *emma* app in real-world conditions.

**Methods:**

The Ecological Mental Momentary Assessment (EMMA) study is an ongoing, longitudinal, interventional, multicenter trial in which patients at high risk for suicide are recruited to test *emma*, an app designed to be used as a self-help tool for suicidal crisis management. Participants undergo clinical assessment at months 0, 1, 3, and 6 after inclusion, mainly to assess and characterize the presence of mental disorders and suicidal thoughts and behaviors. Patient recruitment is still ongoing. Some data from the first 14 participants who already completed the 6-month follow-up were selected for this case report study, which evaluated the following: (1) data collected by *emma* (ie, responses to EMAs), (2) metadata on *emma* use, (3) clinical data, and (4) qualitative assessment of the participants' experiences.

**Results:**

EMA completion rates were extremely heterogeneous with a sharp decrease over time. The completion rates of the weekly EMAs (25%-87%) were higher than those of the daily EMAs (0%-53%). Most patients (10/14, 71%) answered the EMA questionnaires spontaneously. Similarly, the use of the Safety Plan Modules was very heterogeneous (2-75 times). Specifically, 11 patients out of 14 (79%) used the Call Module (1-29 times), which was designed by our team to help them get in touch with health care professionals and/or relatives during a crisis. The diversity of patient profiles and use of the EMA and EMI modules proposed by *emma* were highlighted by three case reports.

**Conclusions:**

These preliminary results indicate that patients have different clinical and digital profiles and needs that require a highly scalable, interactive, and customizable app. They also suggest that it is possible and acceptable to collect longitudinal, fine-grained, contextualized data (ie, EMA) and to offer personalized intervention (ie, EMI) in real time to people at high risk of suicide. To become a complementary tool for suicide prevention, *emma* should be integrated into existing emergency procedures.

**Trial Registration:**

ClinicalTrials.gov NCT03410381; https://clinicaltrials.gov/ct2/show/NCT03410381

## Introduction

### Context

According to the World Health Organization (WHO), suicide is the cause of 1 million deaths per year, accounting for nearly 2% of all deaths worldwide; this number should increase to 1.5 million by 2021. The WHO recognizes suicide prevention as a public health priority [1,2]. History of suicide attempts is the most important risk factor of suicide death in the general population and is present in about 40% of suicides [3,4]. Patients who come to the emergency department (ED) following a suicide attempt present an extremely high risk of suicide in the short term [5-7]. People at risk of suicide often do not seek help and do not remain connected to the health service after an attempt [8]. Access to mental health care is inversely correlated with the suicide rate [9]. It is reported that less than half of patients at risk of suicide are in contact with the health system, including mental health services [10].

### Ecological Momentary Assessment

Real-time emotional, behavioral, and psychological assessments could improve the identification of high-risk individuals who require rapid interventions. Ecological momentary assessment (EMA), also commonly defined as the experience sampling method, allows for the collection of longitudinal fine-grained data as they occur in the real world. It gives an accurate picture of the patient’s symptoms [11] and reduces the impact of self-report response bias, thus leading to a better appreciation of the temporal dynamics of suicide risk [12].

Typically, suicidal ideation is episodic, with a quick onset [13] and short duration (ie, shorter than an hour) [14]. Suicidal thoughts vary dramatically among individuals [15], and fluctuating or persistent suicidal thoughts are associated with the risk of different suicidal behavior types [14]. Suicide attempts can occur in response to a rapid increase in suicidal thoughts within a very short time (ie, 1 day) [13,16,17]. These observations highlight the importance of not relying on intermittent assessments of suicidal ideation for clinical decision making, such as hospital discharge [13,18]. Mobile health (mHealth) interventions are a promising way to assess these fluctuations in real time.

### Ecological Momentary Intervention for Suicide Prevention

Digital tools could increase the effectiveness of these prevention strategies. Indeed, apps are affordable and ubiquitous and can be used in any situation, particularly during a crisis [19]. The WHO recommends them for people at risk of suicide [1] because they offer new opportunities to overcome some of the help-seeking barriers they face [20-23] and enhance safety planning [24] in response to dynamic suicidal processes in real time [13]. Apps that specifically target suicidal behavior and propose interactive and proactive content constitute an effective prevention strategy [25-28]. Despite this scientific evidence, a literature review [29] identified very few suicide-specific apps (n=24) that include safety planning (n=14) and that directly allow the user to seek support (n=13). Feasibility studies of these apps report a significant reduction of suicidal ideation in patients and a significative augmentation of suicide-related coping [30]. However, potentially harmful content might encourage self-harm and suicide [31], and many of the existing apps for suicide prevention have not been scientifically validated [22,29]. The field of e-mental health is particularly active, producing new apps at an extremely fast pace; therefore, it is crucial to regulate this field, especially for suicidology.

Yet, it should be possible to assess and prevent suicidal behaviors in real time in high-risk patients using digital tools. To test this hypothesis, our multidisciplinary team developed *emma*, an app to monitor the psychological, emotional, and social fluctuations of patients in their daily life. In addition to EMA, the app includes interactive and customized ecological momentary intervention (EMI) modules for suicide prevention. This app is currently being tested in an ongoing trial: the Ecological Mental Momentary Assessment (EMMA) study. This article presents a descriptive analysis of selected patients at high risk of suicide to obtain insights into the implementation of an mHealth-based suicide risk assessment and prevention procedure in real-life conditions.

The main objective of this study was to give a quantitative description of *emma* use by a sample of individuals at high risk of suicide in real-life conditions.

The secondary objectives were as follows:

1. To describe typical user profiles of the *emma* app.
2. To describe relevant qualitative elements from the interviews of participants who completed the 6-month study.

## Methods

### Study Design

The EMMA study is an ongoing, prospective, longitudinal, interventional multicenter trial, involving four French university hospitals in Montpellier, Lille, Brest, and Créteil, completed by a qualitative study. The protocol was registered at ClinicalTrials.gov (NCT03410381) on January 18, 2018; was authorized by the French National Agency for Medicines and Health Products Safety (*Agence Nationale de Sécurité du Médicament et des Produits de Santé* [ANSM]) on November 30, 2017; and approved by the Est IV Ethical Committee for the Protection of Patients on October 10, 2017.

### Participants

The EMMA study planned to recruit 100 patients from EDs and mental health departments. Patients are included after a suicide attempt (<8 days) and/or if they have suicidal ideation (ie, score ≥2 out of 3 for item 18 on suicidal ideation of the 30-item Inventory of Depressive Symptomatology: Clinician scale). The other inclusion criteria are as follows: aged 18 years or older, provided a signature on the informed consent form, and possess a smartphone (iOS or Android). Exclusion criteria are as follows: refusal to participate, under guardianship, protected by law, deprived of liberty, not affiliated with a social security system, in a period of exclusion from other trials, and unable to understand the study. The recruited participants will not receive any remuneration for their participation in the study.

### Procedure

At inclusion (ie, month 0 [M0]), a psychiatrist performs the first interview to ensure that the patient meets the eligibility criteria and to obtain the informed written consent. Four clinical assessments are conducted: at inclusion and at months 1, 3, and 6 (M1, M3, and M6). Clinical data are collected using clinician-rated questionnaires—approximate durations are 1 hour and 30 minutes at M0, then 30 minutes at M1, M3, and M6—and self-rated questionnaires—approximate duration ranges from 1 hour to 1 hour and 30 minutes. These questionnaires are listed in Multimedia Appendix 1 [32-48].

Satisfaction concerning the app is evaluated in three distinct ways:

1. Questions about the usefulness and satisfaction of the app administered every month via *emma* (eg, “This month, did you find *emma*: easy to use/intrusive/useful/efficient,” rated using a Likert scale from 0 to 10; approximate completion time is 5 minutes).
2. Standardized self-administered questionnaire—Mobile App Rating Scale [32]—at the end of the study.
3. A qualitative semistructured interview led by a social sciences researcher in mental health proposed to 25 patients to assess the participants’ subjective experiences (approximate duration is 1 hour).

At inclusion, a member of the research team helps patients to install, configure, and personalize the app, particularly to define the elements of their safety plan. Patients are asked to use *emma* for 6 months. Data collected during the assessments made by clinicians, as well as data and metadata resulting from *emma* use, are encrypted and stored in a secure server. All these data will be used to develop the algorithm to predict suicidal risk (see Multimedia Appendix 2). Considering the crucial issue of health data privacy and security, multilevel technical and organizational safeguards were put in place. The app is secured by a password, as requested by the co-designer patients, and data are anonymized, encrypted, and stored in a secure server to prevent unauthorized data disclosure or breach, as recommended by the European General Data Protection Regulation.

### Outcome Measures

The principal outcome measure of this preliminary analysis was the quantitative description of app use: completion rates of daily and weekly EMA questionnaires, frequency of use of the prevention modules, and number of calls made through *emma* to relatives and health care professionals in case of emergency.

The secondary outcome measures were as follows:

1. Occurrence of a suicide event (ie, suicide attempt, hospitalization for suicidal ideation, and intensity of suicidal ideation level) during the study period.
2. Quantitative description and app use timeline in a few selected patients to illustrate different users’ profiles.
3. Qualitative analysis of selected interviews of participants who completed the 6-month follow-up.

### Emma Design

*Emma* is a smartphone app developed for the assessment, prevention, and, ultimately, prediction of suicidal behaviors. It was designed by integrating evidence-based suicide prevention strategies and recommendations for the development of apps in the field of mental health [19,49]. *Emma* design was based on data from previous suicide-specific apps described in the theoretical literature and on practical data obtained by our research team by testing the available suicide-related apps. *Emma* was developed for Android and iOS for wide usage.

*Emma* was conceived using a participatory design approach that included individuals with lived experience of suicidal thoughts and behaviors as equal partners of our professional multidisciplinary team—researchers, psychiatrists, psychologists, sociologists, computer scientists, engineers, and data scientists—from start to finish, as recommended [50-52]. This ensured that *emma* met scientific and technical standards as well as the patients’ needs as stated in the literature [53-55]. To involve the targeted users, a methodology based on focus groups was implemented according to the method proposed by Krueger and Casey [56]. Early in the development process, the involved patients stressed the importance of having a secure password to open the app; they also contributed to the choice of the name “*emma,*” which they wanted to be not stigmatizing and without any mention of psychiatry or suicide. A group of co-designer patients (n=5) and clinical staff (n=5) tested the app extensively for 3 months, and their feedback was taken into account in an iterative way by the developers at the Laboratory of Informatics, Robotics, and Microelectronics of Montpellier to improve the app. Particularly, the developers made sure that the notifications asking users to fill in the EMA questionnaires did not lead to interruption or disruption in the users’ daily activities in order to maximize *emma* acceptability, use, and validity, as recommended [54,55].

### Emma Contents

*Emma* was designed to be used as a self-help tool for suicidal crisis management. Patients are invited to identify the following:

1. Their warning signs (eg, negative feelings and problematic behaviors).
2. Their individualized coping strategies (eg, *Breathing Space*: an audio awareness guide made specifically for *emma* by a psychiatrist expert in suicidal behavior).
3. Their distraction activities in an *Emotion Regulation Module* (eg, favorite places and activities and libraries of music and images that might help to connect with the patient’s reasons for living [19]).
4. Their social support (eg, collating the contact details of the patient’s social network, mental health professionals, and other crisis resources). This module promotes connectedness, a major protective factor in suicide prevention [21].

A *Call Module* allows the patient to contact, depending on the severity of his or her condition, (1) the relatives he or she has identified, (2) the ED that is following that patient, and (3) the Service d'Aide Médicale d'Urgence (SAMU), the French national emergency medical assistance service, which is available 24/7.

Although the safety plan implies restriction of access to lethal means, *emma* does not contain any mention of them because, according to the literature, this can have the opposite effect. Specifically, their identification can facilitate their use through an effect called *cognitive availability* [29]. Examples of *emma* screens are shown in Multimedia Appendix 3.

**Figure 1:**
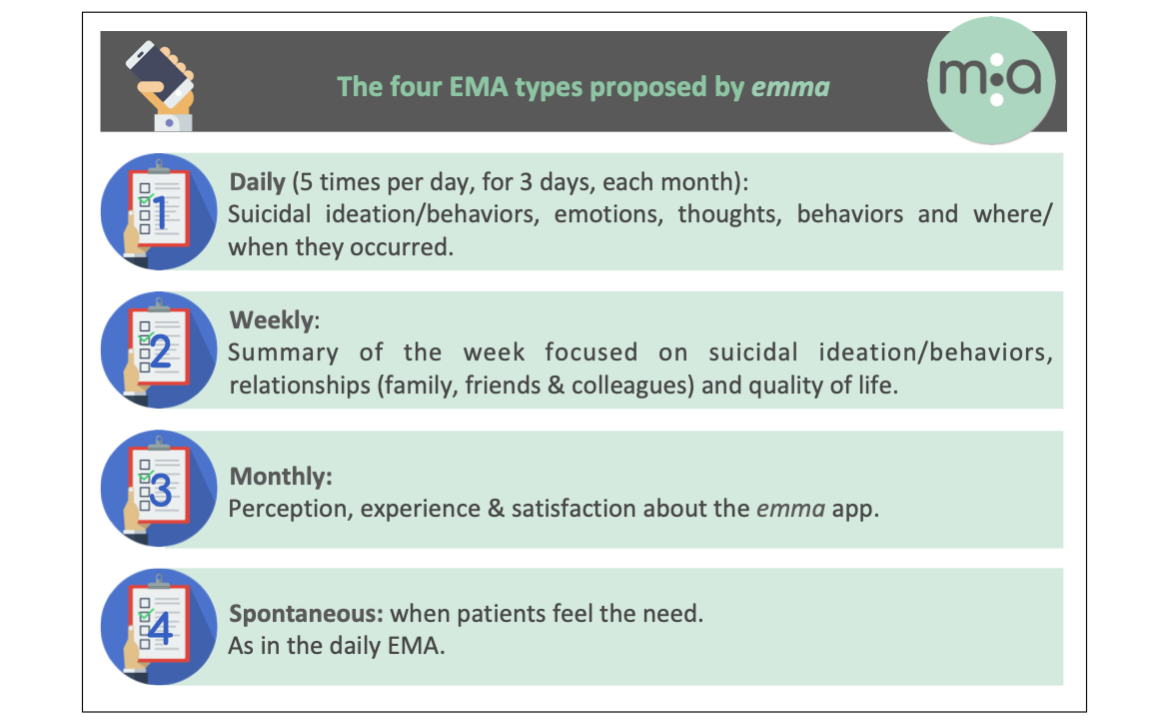
Ecological momentary assessment (EMA) types proposed by *emma*.

*Emma* proposes four brief EMA types: three scheduled evaluations at predetermined frequencies (ie, daily, weekly, and monthly) and one spontaneous assessment (see Figure 1). Depending on the questions asked, specific response modalities are provided:

1. Likert scales (from 0 to 10) for questions such as “What is your level of moral pain?”
2. Single or multiple-choice check boxes for questions such as “What is your level of mental pain?”; for instance, *I am keeping myself busy to avoid thinking*, *Nothing*, *I am ruminating*, *I am doing something I enjoy*, *I am working*, and *I am doing household chores*.
3. Writing a free-text answer for questions such as “What is the experience that has affected you the most since this morning?”

**Figure 2:**
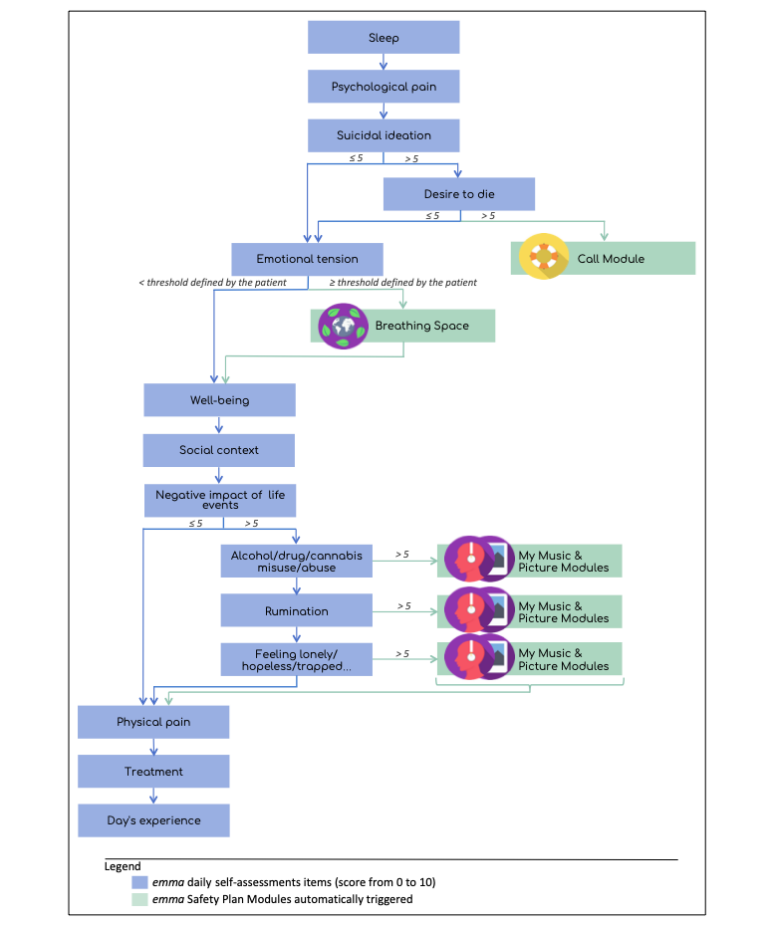
Algorithm for automatic ecological momentary intervention (EMI) triggering according to the ecological momentary assessment (EMA) answer thresholds.

During the focus group process, alarm thresholds were defined on the basis of the patient’s answers to critical questions that led to the automatic suggestion of adapted EMI modules (see Figure 2). For each EMI module, the app presents a list of suggestions that can be modified (ie, content and order) by each patient (eg, for *Favorite activities*, responses can be *Taking a bath*; *Going to the cinema, theatre, museum, or a concert*; *Going for a walk*; *Reading*; *Spending time with good friends*; *Doing sports*; etc). Modules are designed to be adaptable to the user’s state, needs, and strategies.

### Global Descriptive Analysis and Case Reports

The use of *emma* was described by computing (ie, mean [SD] and min and max) the number of answers and frequency of completion of the daily and monthly questionnaires. The overall use of the different *emma* modules (ie, *Call Module, Emotion Regulation Modules, Breathing Space Module, Pictures,* and *Music)* was also computed. Finally, the use of EMA (ie, spontaneous and scheduled suicidal ideation assessments) and EMI (ie, *Call Module* and *Emotion Regulation Module* of the safety plan) by 3 patients during the 6 months of the study was described relative to (1) their suicidal ideation scores (ie, sums of items 7-11 of the Columbia-Suicide Severity Rating Scale [C-SSRS]) assessed during the scheduled visits (ie, M0, M1, M3, and M6) and (2) their admissions to the ED for suicide attempts or suicidal crises.

## Results

### Baseline Assessment

**Figure 3:**
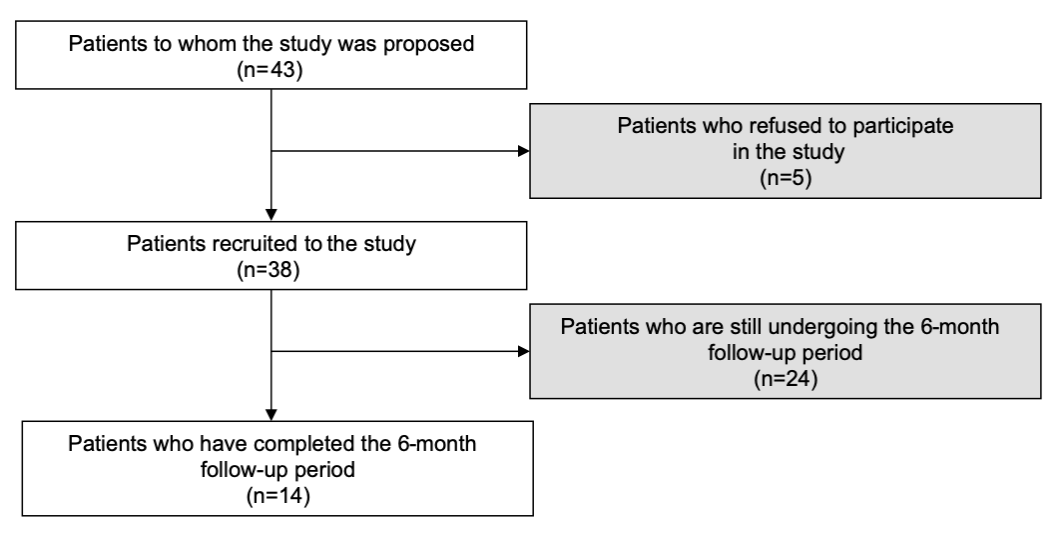
Flowchart of patient selection.

From May 2018 to March 2019, participation in the EMMA study was proposed to 43 patients at risk for suicide (ie, recent attempters or current ideators) who were admitted to the ED or hospitalized in a postemergency department; 38 (88%) agreed to participate. A total of 5 patients refused to participate because they considered the study duration of 6 months to be too long. The analysis in this study included 14 patients who have already completed the study (see Figure 3). The patients’ sociodemographic and clinical data are reported in Tables 1 and 2, respectively. These first *emma* users were mostly women (12/14, 86%), with a mean age of 34 years (SD 13, min-max 18-57). All participants presented at least one mental disorder, according to criteria from the Diagnostic and Statistical Manual of Mental Disorders, 5th Edition (DSM-5), and most of them presented more than one (ie, up to five comorbid disorders); 6 out of 14 patients (43%) were recent suicide attempters (≤8 days), and they all had severe suicidal ideation (ie, mean C-SSRS score 22 [SD 4], min-max 15-25).

During the study, 4 out of 14 (29%) patients were admitted to the ED with suicidal ideation and/or a suicide attempt one to three times, and 1 (7%) patient made a suicide attempt without ED admission (see Multimedia Appendix 4).

A total of 2 patients out of 14 (14%) left before the first follow-up visit: one was excluded because she was restrained, and the other one withdrew from the study.

**Table 1:** Sociodemographic data of participants.

Patient No.	Sex	Age (years)	Marital status	Number of children	Cohabitation	Professional situation
1	Female	24	Single	0	Lives alone	Working (stable)
2	Female	18	Single	0	Lives with family	Student or in training
3	Female	27	In a relationship	0	Lives alone	Volunteer
4	Female	33	In a relationship	2	Lives with family	Working (stable)
5	Female	40	In a relationship	2	Lives with family	Working (precarious)
6	Female	46	Separated	3	Nonfamily cohabitation	Without activity, at home
7	Female	30	Single	0	Lives alone	Disability
8	Male	48	In a relationship	2	Lives with family	Sick leave
9	Female	57	Separated	4	Lives with family	Disability
10	Female	48	In a relationship	2	Lives with family	Sick leave
11	Female	21	In a relationship	0	Nonfamily cohabitation	Student or in training
12	Female	23	Single	0	Lives alone	Student or in training
13	Male	18	Single	0	Lives with family	Student or in training
14	Female	48	Separated	1	Lives alone	Working (stable)

**Table 2:** Clinical data of participants.

Patient No.	Current DSM-5^a^ diagnoses	Borderline personality	Suicidal risk in the near future according to the DSM-5	Suicidal ideation intensity^b^	Number of suicide attempts	Number of severe or violent suicide attempts^c^	Family history of suicide attempt	Family history of suicide
1	Major depressive disorder	No	Yes	22	2	N/A^d^	No	No
2	Major depressive disorder Agoraphobia Social phobia Anorexia	Yes	Yes	25	2	N/A	No	No
3	Major depressive disorder Agoraphobia Social phobia Bulimia	Yes	No	25	30	Violent (n=2) Severe (n=1)	Yes	No
4	Major depressive disorder	No	Yes	25	2	N/A	Yes	No
5	Depressive episode Severe alcohol and substance-related disorder Bulimia Bipolar disorder II	No	No	23	1	N/A	Yes	Yes
6	Major depressive disorder Severe alcohol-related disorder	No	Yes	15	2	N/A	No	No
7	Depressive episode Generalized anxiety disorder Bipolar disorder I	No	Yes	25	55	Violent (n=15)	No	No
8	Depressive episode Social phobia Severe alcohol-related disorder Generalized anxiety disorder Bipolar disorder I	No	No	25	1	N/A	Yes	No
9	Major depressive disorder Agoraphobia Posttraumatic disorder Bipolar disorder II	No	Yes	25	8	Severe (n=1)	No	No
10	Major depressive disorder	No	Yes	24	1	N/A	Unknown	Yes
11	Generalized anxiety disorder	No	Yes	16	1	N/A	Yes	No
12	Major depressive disorder Agoraphobia Social phobia Bulimia	No	Yes	19	0	N/A	Yes	Yes
13	Depressive episode Social phobia Generalized anxiety disorder	Yes	Yes	20	3	N/A	No	No
14	Major depressive disorder	No	No	18	1	N/A	No	No

^a^DSM-5: Diagnostic and Statistical Manual of Mental Disorders, 5th Edition.

^b^Sums of items 7-11 of the Columbia-Suicide Severity Rating Scale (C-SSRS): scores range from 0 to 25.

^c^A violent suicide attempt was defined by the method of violence: weapons, hanging, jumping from a height, traffic, drowning, or immolation. A severe suicide attempt was defined as requiring hospitalization in the intensive care unit.

^d^N/A: not applicable; suicide attempts, if applicable, were not severe or violent.

### Main Outcome

The completion rates were higher for the weekly versus daily EMAs (mean 24% [SD 34], min-max 25%-87%; mean 4% [SD 12], min-max 0%-53%, respectively). Moreover, they were extremely heterogeneous among participants with a sharp decrease over time. Most patients (10/14, 71%) filled in the questionnaires spontaneously at times of crisis (mean 7 times [SD 12], min-max 1-39 times).

Similarly, use of the EMI *Safety Plan Modules* varied among patients (mean 11 times [SD 19], min-max 2-75 times). Most patients (11/14, 79%) used the *Call Module* (mean 7 times [SD 8], min-max 1-29 times) to get in touch with the health care system and/or family and friends during a crisis. Most patients (10/14, 71%) called the SAMU (mean 4 times [SD 5], min-max 1-15 times), 8 (57%) called their relatives (mean 3 times [SD 2], min-max 1-7 times), and 7 (50%) called their ED (mean 2 times [SD 2], min-max 1-7 times). About half of the patients (8/14, 57%) used one of the *Emotion Regulation Modules* (mean 10 times [SD 15], min-max 1-46 times). Specifically, 8 out of 14 (57%) patients listened to the *Breathing Space Module* (mean 3 times [SD 1], min-max 1-5 times), while only 4 (29%) looked at *Pictures* (mean 12 times [SD 19], min-max 1-41 times) and 2 (14%) listened to *Music* (mean 4 times [SD 3], min-max 2-7 times).

### User Profiles

Out of 14 users, 3 (21%) are described in detail to better illustrate the different *emma* uses. These patients were chosen to reflect the diversity of the patients' clinical profiles and of *emma* use (ie, completion rate of the scheduled EMAs and use of the *Safety Plan Modules*).

#### Patient 7

**Figure 4:**
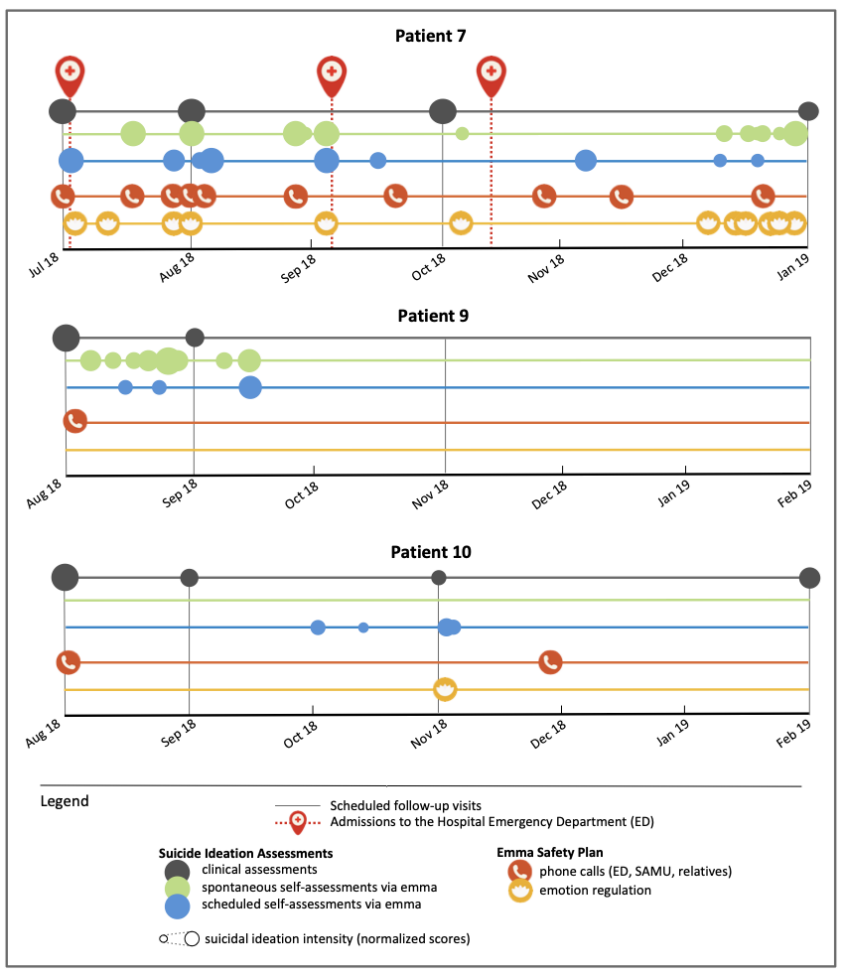
Case reports for Patients 7, 9, and 10. SAMU: Service d'Aide Médicale d'Urgence.

Patient 7 is a 30-year-old single woman living alone, without children, unemployed, and receiving a disability living allowance for adults (see Table 1). At inclusion, she had major depressive disorder, generalized anxiety disorder, and bipolar disorder I, according to the DSM-5. She had 55 previous suicide attempts, of which 15 were violent, and a maximal suicidal ideation intensity at inclusion (C-SSRS score of 25 out of 25) (see Table 2). Therefore, she was at very high risk, and the period following hospitalization is known to be a particularly high-risk time for recurrence, as confirmed by her three admissions to the ED during the study period (see Figure 4). The first admission for suicidal ideation occurred just after her hospital discharge. She presented maximal suicidal ideation intensity that was ecologically assessed via *emma*; she used the *Call Module* just before ED admission and an *Emotion Regulation Module* just after. Two months later, in addition to the scheduled EMA, she filled in the questionnaires spontaneously several times with very high suicidal ideation scores. These spontaneous self-assessments were immediately followed by the use of the *Call Module* with a sharp decrease in suicidal ideation. Nevertheless, suicidal ideation rapidly increased again, leading to a new admission to the ED. About a month later, despite the lower suicidal ideation score at the spontaneous self-assessment and the use of the *Emotion Regulation Module*, she was admitted to the ED for an aborted suicide attempt by hanging.

During the study period, Patient 7 completed 66 EMA self-assessments. Specifically, she completed only 4% of the scheduled daily questionnaires due to technical problems (ie, notifications not received) and about 75% of the weekly questionnaires; she frequently filled in the questionnaires in a spontaneous way (39 times), particularly at specific times, without a decrease in frequency during the study period. She often used the *Call Module* (29 times), particularly to contact the SAMU (15 times), followed by the ED (7 times) and relatives (7 times). She also looked at *Pictures* (41 times) and listened to the *Breathing Space Module* (5 times).

A qualitative longitudinal analysis showed that the EMA made via the app allowed for the capture of suicidal ideation fluctuations that were not highlighted by the clinical follow-up visits (see Figure 4). While the first three clinical evaluations (ie, M0, M1, and M3) were stable, the assessments performed via *emma*, both spontaneous and scheduled, showed fluctuations in intensity over time. The assessments also showed a regular use of the *Safety Plan Modules*, at least one module per month, the frequency of which increased at specific times. For instance, in July 2018, the high intensity of suicidal ideation evaluated ecologically by *emma* corresponded to an increased use of the *Call Module*. December 2018 was characterized by many spontaneous EMA completions, lower suicidal intensity, and frequent use of the *Emotion Regulation Module*. This suggests that *emma* alarm thresholds triggered EMIs that were adapted to her condition severity.

#### Patient 9

Patient 9 is a 57-year-old single woman living with her four children. She is unemployed with a disability living allowance for adults (see Table 1). At inclusion, she had major depressive disorder, agoraphobia, posttraumatic disorder, and bipolar disorder II, according to the DSM-5. At the first clinical evaluation, suicidal ideation intensity was very high (C-SSRS score of 25 out of 25) with suicide risk in the near future. She already committed eight suicide attempts, including one severe attempt (see Table 2). During the study, the clinician reported a decrease in suicidal thoughts at M1, and then absence of suicidal thoughts at the M3 and M6 visits.

Patient 9 rarely filled in the daily EMAs (10%), but she completed 81% of the weekly EMAs and filled in EMAs spontaneously 29 times. She completed 100% of the monthly questionnaires about her perception, experience, and satisfaction about *emma* use. The longitudinal qualitative analysis (see Figure 4) showed that from month 2 after inclusion, both the self-assessments made with *emma* and the hetero-assessments made during the visits did not detect any suicidal ideation. Patient 9 used her safety plan only once at the very beginning of the study when she still had suicidal thoughts.

#### Patient 10

Patient 10 is a 48-year-old woman living with her husband and their two children. When included in the study, she was on sick leave (see Table 1). She had major depressive disorder and suicidal risk in the near future, according to the DSM-5, and very high suicidal ideation intensity (C-SSRS score of 24 out of 25). She reported one suicide attempt and a family history of suicide (see Table 2). Patient 10’s suicidal ideation decreased at the M1 and M3 follow-up visits, but was higher again at the M6 visit.

Patient 10 never filled in questionnaires spontaneously, filled in the daily EMAs only once, and did not fill in the weekly EMAs often (35%). She completed only one monthly questionnaire about her *emma* experience. She used her safety plan several times, mainly to call her relatives (3 times), the SAMU (3 times), and the ED (2 times). She also listened to the *Breathing Space Module* (3 times).

Patient 10 reported suicidal thoughts via *emma* only during a brief period—in October and early November 2018—and during that period she used the *Emotion Regulation Module*, also only once (see Figure 4). Self-assessment may have helped her to become aware of her condition and use the tools that *emma* offers to regulate her emotions. This patient used the *Call Module* twice: immediately after her inclusion in the study, in August 2018, when she had severe suicidal thoughts and suicide risk, and then at the end of November 2018 when no suicidal thought was reported via *emma* at that time.

### Qualitative Study

The initial qualitative feedback from the participants collected during the qualitative study, using semidirected interviews, was very positive. It emphasized the support and the connectedness dimensions allowed by the app. During the co-design process, patients proposed to give the app a female name to personify it. The first users interviewed seemed to appreciate this, as stressed by a patient: “*Emma* is like having a companion.”

This *digital companion* seemed to help reduce the feeling of loneliness, as expressed by a patient: “We have the feeling that we are not alone, that the software supports us.” Moreover, this support comes at a critical time, after hospital discharge. To our knowledge, this period of very high suicide risk [15], when patients may experience painful loneliness in contrast to the time in hospital where they are followed by the health care team 24 hours per day, has not been studied much. One patient stated, “Thanks to *emma*, I did not feel alone when I left the hospital. I know *emma* less than my relatives, but I can tell her more.” *Emma* also appeared to be a support, offer autonomy, and be an empowerment tool by helping people to use their own resources, as expressed by a patient: “*Emma* is an appointment with oneself. It is the memory of the patient: I know who to call.” This empowerment was facilitated by the possibility to personalize the app, as expressed by a patient: “It is important that we can each fill in the things that affect us and the things that impact us, because we are all different and it allows targeting everyone specifically; it is good because it helps us deep down.” These first qualitative interviews highlighted the interest of the co-design process that enabled patients to appropriate the digital tool. During the design phase, a patient indicated that he thought three apps were needed: “When we are doing well, not well, and not well at all.” Therefore*, emma* was designed to be as adaptable as possible. For example, it can offer *Emotion Regulation Modules* if the patient shows tension, and it can offer crisis *Call Modules* that are graduated according to the crisis severity (ie, to relatives, the ED, and, finally, the SAMU). The tailored adjustment of the technology-delivered program is also in line with evidence-based recommendations for mental health, as expressed by a patient: “The app can recommend specific solutions to each user’s specific problems” [19].

## Discussion

### Principal Findings

These preliminary descriptive results of the use of a suicide prevention app indicate that this type of digital tool could be accepted and used by patients at high risk of suicide. The study participation rate (88%) was very good compared with other studies. For example, Hallensleben et al reported that 47% of inpatients with unipolar depression agreed to complete EMA [18]. Our preliminary results show an encouraging patient acceptance rate and highlight the great inter- and intraindividual diversity of app usage patterns (ie, EMA questionnaires’ completion rate and/or *Safety Plan Modules* use). Patients have different needs and different clinical and digital profiles; therefore, a highly scalable, interactive, and customizable app is required.

The connectedness philosophy implemented in *emma* is considered a strong protective factor for suicidal behaviors [57,58]. *S*ubjectively perceived and effectively received social supports improve mental and physical well-being [59,60]. *Emma* has the potential to act on the quality of patients' social ties and their social pain that seems to be involved in suicidal crises [61]. Patients expressed the importance to feel supported by the software and to have the opportunity to tell *emma* things they would not dare to say to their relatives, to protect them and/or out of fear of the stigma that their confidences could generate.

Concerning the co-design methodology, patients were consulted very early and at all stages of this project through focus groups for the app design, then through feedback during prototype testing, and, finally, through qualitative interviews with *emma* users. However, as patients with suicidal behavior are a high-risk population, it is important to ensure that their participation in research respects the safety principle. Therefore, a trust-based exchange framework that was flexible enough to adapt to fluctuations in their condition was put in place. When carefully implemented, the participation of patients as partners can be a factor of empowerment and self-esteem restoration [62].

Our analysis showed heterogeneous use and engagement with the *emma* app, most often underuse relative to our expectations. This could be interpreted as poor adherence to the app [63,64] and was partly caused by technical problems experienced by the first participants (eg, notifications did not appear at the beginning of the study). *Emma* use by Patient 10 suggests that the app can be useful at times of crisis, even when the scheduled questionnaires are not completed. Besides the simple quantitative measure of app use, it was also important to evaluate the users’ subjective experiences [65] based on mixed methods, as recommended [66]. This should allow for the identification of different user profiles and for the development of tailored prevention strategies [67].

### Conclusions

Data on immediate and long-term risk of suicide are extremely sparse and based on measures with poor temporal resolution [68,69]. *Emma* is a great opportunity to capture the dynamics of suicidal ideas [67] and their translation into action in a contextualized way that allows for a much more nuanced view of variables over time [70]. Hopefully, this digital tool might lead to scientific and clinical advances and will allow for the identification of high-risk periods and prediction of imminent risk, which are extremely challenging at the moment [71]. These fine-grained digital assessments and predictive mHealth-based interventions are promising tools for suicide prevention [55], because they represent an unprecedented opportunity to act at multiple levels through targeted, scalable, and contextualized micro-interventions [72]. They might allow for the proposal of just-in-time adaptive interventions, defined by Nahum-Shani et al [73] as the *right support* (eg, type and intensity) *at the right time* [31]. The challenge is now to integrate such digital interventions into the existing health care systems [52]. For instance, *emma* could be integrated into ED procedures and become a complementary health care tool. In the patient’s pocket, *emma* could provide individualized support when needed. The app could improve coordination among the different services (ie, the ED, crisis centers, hospital services, outpatient services, and general practitioners). However, the smooth and optimal integration of such digital tools in patient care requires health care professionals’ support. Indeed, they should not perceive these tools as disruptive elements in their daily clinical practice [74,75], but as a support to improve the therapeutic relationship within a well-defined ethical, social, and legal framework.

## Appendix

Multimedia Appendix 1 List of the questionnaires filled in by the patient or by the clinician during the four visits.

Multimedia Appendix 2 Visual description of the study. Study protocol: visits (month 0 [M0], month 1 [M1], month 3 [M3], and month [M6]) and real-life use of emma by the recruited patients. At the end of the study, the data collected directly by the app and the clinical data collected during the visits are integrated to develop the suicide risk prediction algorithm.

Multimedia Appendix 3 Emma screenshots.

Multimedia Appendix 4 Adverse events during the follow-up.

## Abbreviations

ANSM: Agence Nationale de Sécurité du Médicament et des Produits de Santé (French National Agency for Medicines and Health Products Safety)
C-SSRS: Columbia-Suicide Severity Rating Scale
DSM-5: Diagnostic and Statistical Manual of Mental Disorders, 5th Edition
ED: emergency department
EMA: ecological momentary assessment
EMI: ecological momentary intervention
EMMA: Ecological Mental Momentary Assessment
M0: month 0 (inclusion)
M1: month 1
M3: month 3
M6: month 6
mHealth: mobile health
NERB: Neurophysiology of Repetitive Behaviors
SAMU: Service d'Aide Médicale d'Urgence
WHO: World Health Organization
