# Supplementary material for: A Digital Companion, the Emma App, for Ecological Momentary Assessment and Prevention of Suicide: Quantitative Case Series Study
Source: JMIR Mhealth Uhealth. 2020 Oct 9;8(10):e15741. doi: 10.2196/15741 (PMC7584985; doi:10.2196/15741)
Supplement: Multimedia Appendix 1 [file mhealth_v8i10e15741_app1.docx]

**Multimedia Appendix 1: List of the questionnaires filled in (by the patient or by the clinician) during the four visits**

| **Questionnaire** | | **Assessed dimensions** | **M0** | **M1** | **M3** | **M6** |
| --- | --- | --- | --- | --- | --- | --- |
| **Clinician-rated questionnaires** | Mini International Neuropsychiatric Interview (Mini 7) [33] | Lifetime psychiatric diagnoses (DSM 5) | X |  |  |  |
|  | Inventory of Depressive Symptomatology  (IDSC-30) [34] | Depression | X | X | X | X |
|  | Columbia–Suicide Severity Rating Scale (CSSRS) [35] | Suicidal spectrum | X | X | X | X |
|  | Risk Rescue Rating Scale-(RRRS) [36] |  | X | X  If new SA | X  If new SA | X  If new SA |
|  | Suicidal Intent Scales (SIS) [37] |  | X | X  If new SA | X  If new SA | X  If new SA |
| **Self-rated questionnaires** | Quick Inventory of Depressive Symptomatology (QIDS) [34] | Depression | X | X | X | X |
|  | Childhood Trauma Questionnaire (CTQ) [38] | Childhood abuse | X |  |  |  |
|  | University of Laval's loneliness scale (ESUL) [39] | Loneliness | X | X | X | X |
|  | Index of social isolation (ISI) [40] | Social isolation | X | X | X | X |
|  | Beck hopelessness scale (BHS) [41] | Hopelessness | X | X | X | X |
|  | Reasons for Living Inventory  (RFLS) [42] | Reasons for living | X | X | X | X |
|  | Impulsive Behavior Scale  (UBS) [43] | Impulsivity | X |  |  |  |
|  | Difficulties in Emotion Regulation Scale (DERS) [44] | Emotion regulation | X |  | X | X |
|  | Relationship Scales Questionnaire (RSQ) [45] | Attachment | X |  |  |  |
|  | Interpersonal Needs Questionnaire-Revised  (INQ-R) [46] | Interpersonal Needs | X |  | X | X |
|  | The Short Form (36) Health Survey (SF-36) [47] | Quality of life | X |  | X | X |
|  | Life regard index (LRI) [48] | Meaning in life | X |  | X | X |
|  | Mobile Application Rating Scale (MARS) [32] | Satisfaction of application |  |  |  | X |
