# Supplementary figures and images for: A Digital Companion, the Emma App, for Ecological Momentary Assessment and Prevention of Suicide: Quantitative Case Series Study
Source: JMIR Mhealth Uhealth. 2020 Oct 9;8(10):e15741. doi: 10.2196/15741 (PMC7584985; doi:10.2196/15741)

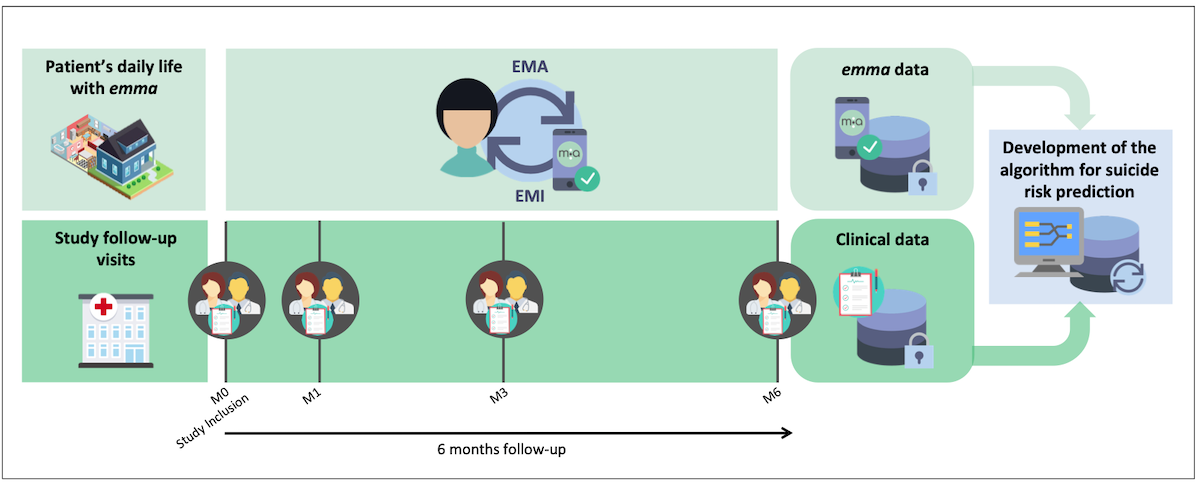

Supplement: Multimedia Appendix 2 [file mhealth_v8i10e15741_app2.png]

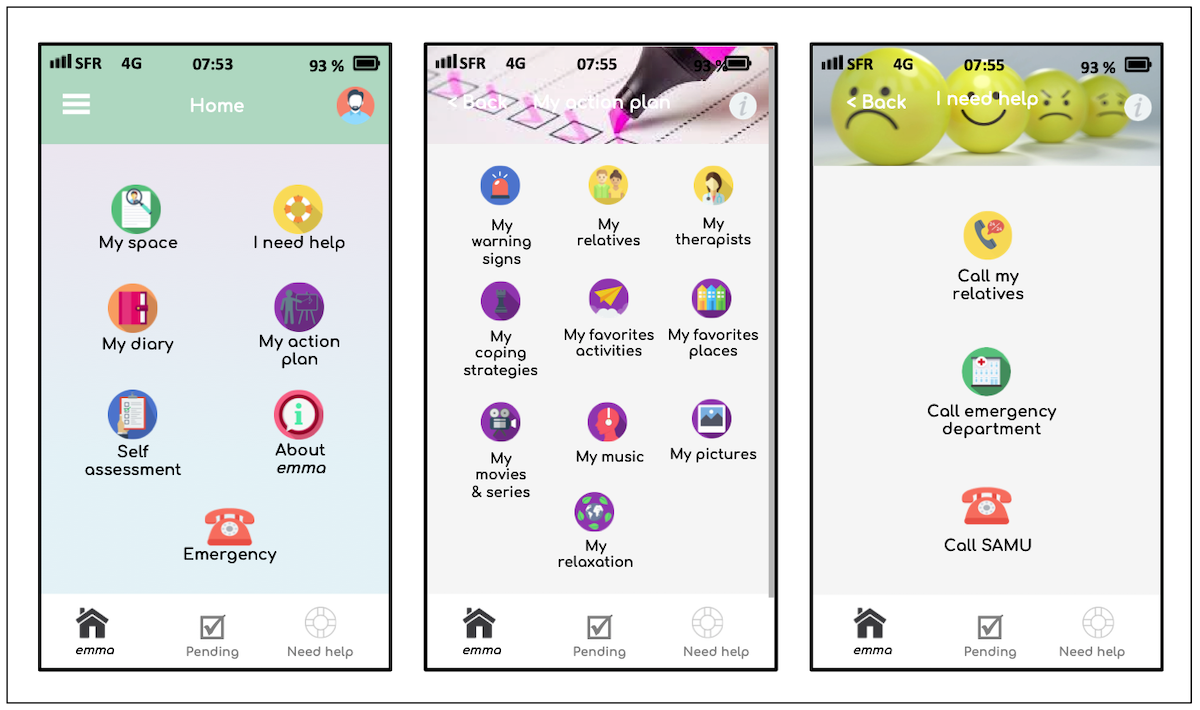

Supplement: Multimedia Appendix 3 [file mhealth_v8i10e15741_app3.png]
