# Supplementary material for: A Digital Companion, the Emma App, for Ecological Momentary Assessment and Prevention of Suicide: Quantitative Case Series Study
Source: JMIR Mhealth Uhealth. 2020 Oct 9;8(10):e15741. doi: 10.2196/15741 (PMC7584985; doi:10.2196/15741)
Supplement: Multimedia Appendix 4 [file mhealth_v8i10e15741_app4.docx]

**Multimedia Appendix 4.** Adverse events during the follow-up

| Patient | Number of suicide attempts | Aborted suicide attempts | Number of emergency department visits | Premature study stop |
| --- | --- | --- | --- | --- |
| 1 |  |  | 1 (road accident) |  |
| 2 | 1 (voluntary drug ingestion)  1 (bladed weapon) |  | 2 (suicide attempts) | Exclusion (deprived of liberty) before visit 1 |
| 3 |  |  | 3 (suicidal ideation) |  |
| 4 |  |  |  |  |
| 5 |  |  |  |  |
| 6 |  |  |  |  |
| 7 |  | 1 (hanging) | 3 (suicidal ideation) |  |
| 8 |  |  |  |  |
| 9 |  |  |  |  |
| 10 |  |  |  |  |
| 11 |  |  |  |  |
| 12 | 1 (voluntary drug ingestion) |  |  |  |
| 13 |  |  |  |  |
| 14 |  |  |  | Withdrawal before visit 1 |
